# Dr. Farrar’s Second Volume

**Published:** 1897-06

**Authors:** 


### DR. FARRAR'S SECOND VOLUME.

The second volume of this great work of Dr. Farrar on "Irregularities of the Teeth and their Correction" is nearing completion, and will be published in the early fall.

The writer had recently the pleasure of inspecting it in advance sheets, and in all its details it met his expectations. It will be a work about equal in size to the first volume, but differs from this in that it is confined entirely to practical illustrations of cases in the author's practice, as well as that of others. It is needless to say that this covers about everything conceivable in malposed teeth.

The text is profusely illustrated in Dr. Farrar's well-known style; and this, with concise descriptions, in clear type, will enable all to follow the methods and repeat the operations without difficulty.

When the three volumes are completed, as contemplated by the author, they will constitute a work unquestionably the most elaborate, as well as the most practical, upon the subject of irregularities attempted in this century, and it is doubtful whether it can be improved upon or duplicated in the next. S. G. Blood, 83 and 85 Duane Street, New York City, is the business manager for the author.

